# A Longitudinal Examination of Young People’s Gambling Behaviours and Participation in Team Sports

**DOI:** 10.1007/s10899-022-10175-x

**Published:** 2022-12-17

**Authors:** Brendan Duggan, Gretta Mohan

**Affiliations:** 1 Higher Education Authority, Dublin, Ireland; 2 Department of Economics, Trinity College, Dublin, Ireland; 3 Economic and Social Research Institute, Sir John Rogerson’s Quay, Whitaker Square, Dublin 2, D02 K138 Ireland

**Keywords:** Gambling, Group membership, Online gambling, Social identity, Team sports, Young people

## Abstract

This paper develops and expands upon social identity theory as an explanation for gambling among youth engaged in team sport. Analysing longitudinal data for over 4500 20-year-olds from the *Growing Up in Ireland* study, reveals that online gambling increased from 2.6 to 9.3% between 17 and 20 years in the cohort, with the increase driven by males. A statistically significant positive association is uncovered between playing team sports and regularly gambling, as well as online gambling behaviour, independent of socio-demographic and other risk factors *for males* but *not for females*. The findings provide support for a dose–response like effect for males, where a longer period of participation in team sports is associated with a higher likelihood of engaging in gambling behaviour compared to shorter periods. Implications of the findings for policy and practice are discussed.

## Introduction

Globally, gambling availability and participation has increased to historically unprecedented levels in the last thirty years (Abbott, 2020). Epidemiological studies tend to find that problem gambling is in the range of 1% to 3% of the population, peaking before the age of 30 years (Abbott, 2020). Since the onset of the COVID-19 pandemic, there is evidence that traditional (offline) modes of gambling have declined, while internet-enabled access to gambling has increased in prevalence (Emond et al., 2022; Riley et al., 2021). Young people are the highest risk cohort of exposure to, and engagement in, online gambling (Gainsbury et al., 2015; King et al., 2010), and young adults may be at a heightened risk of engaging in problem gambling, making this a highly relevant and important area of study. Gambling behaviours have been associated with a range of negative consequences for young people (Blinn-Pike et al., 2010) and as such, the emergence of online gambling has received a considerable amount of attention from parents, advocacy groups, government bodies, and the European Union (Codagnone et al., 2015; European Commission 2012; European Parliament, 2019; Lopez-Fernandez & Kuss, 2019). To inform policies which try to address the issue of young people adopting problematic gambling behaviours, it is important to understand which factors which may be predictive of their gambling activities over time.

Using a large, longitudinal, and nationally representative dataset of young people growing up in Ireland, the aim of this investigation is to shed light on the characteristics, background factors, and behaviours at 17/18 years which predict whether 20-year-olds report engaging regularly in gambling, and gambling via online channels. Using prospective longitudinal data allows tracking of the trajectory towards higher frequency gambling over a prolonged period, improving upon existing cross-sectional studies which constrain conclusions about the development of gambling over time (Bray et al., 2014).

As well as a need for greater longitudinal insights, one area which merits more scholarly attention is that of young people’s sports participation and gambling, particularly since sport is heavily targeted by gambling advertising. Sport has been used a vehicle for the normalisation of gambling (Deans et al., 2017a, 2017b; Thomas et al., 2015), and to portray gambling as intrinsic to the events being gambled on (Lopez-Gonzalez et al., 2018). Sports players and fans have been found to be more likely to gamble (Martin et al., 2016; Nelson et al., 2007), and we conjecture that involvement in team sports may be an apt environment for engagement in such behaviours among young people. For the investigation of this paper, social identity theory (Tajfel & Turner, 1979) is employed and developed upon, to understand young people’s engagement in team sports and gambling. We propose that perceived social norms within sport team structures may play an explanatory role in a hypothesized higher likelihood of gambling behaviour among team sports participants.

This study specifically attempts to answer the following research questions:

1. What is the change in prevalence of regularly gambling and online gambling between 17/18 years and 20 years?
2. Does exposure to a team sports environment in late adolescence lead to a greater likelihood of engagement in gambling?
  If yes, is there a dose–response like relationship between participation in team sports and likelihood of gambling?
3. Are associations between team sports participation and gambling behaviours different across sex?

The findings of this study are of value for several reasons. They will provide a deeper insight into precursors of young people’s gambling behaviours and can advance our knowledge in identifying such activities as a function of involvement in team sports and other pursuits. The use of longitudinal data provides information on past characteristics and behaviours to use as controls for current outcomes, improving upon previous studies dependent on cross-sectional data, and potentially very valuable for identification of effects. Importantly, a detailed understanding of young people’s gambling behaviours will assist parents, educators, and policymakers in developing policies to improve young people’s awareness of factors which contribute to gambling behaviours.

## Literature Review

Internationally, the prevalence of problem gambling among those under 25 years of age demonstrates a high variation; for Europe, Calado et al. (2017) report that prevalence ranged between 0.2% and 5.6%, while Riley et al. (2021) report a variation of between 1.1% and 9.8% globally. The study setting of this research is Ireland, for which a recent national survey estimated that 12% of those those aged 15–25 were at an elevated risk of problem gambling and 0.8% met the criteria for problem gambling (Mongan et al., 2022). For young males, 18% were at an elevated risk, and 1.5% were problem gamblers, a rate three times higher than the population at large. Many academic studies find a higher likelihood of both gambling behaviour and problem gambling among males (see Riley et al., 2021). Studies examining the relationship among adolescents and young adults in terms of gambling find associations with use of alcohol, tobacco, and cannabis (Hammond et al., 2014; Peters et al., 2015; Volberg et al., 2010; Weinberger et al., 2015). A large 33-country European study of 16-year-olds found positive associations between gambling behaviour and both alcohol and tobacco use, but no significant association with cannabis use (Molinaro et al., 2018). Yücel et al. (2015) found that, among the risky behaviours they examined, alcohol use had the strongest longitudinal association with later higher frequency gambling, and Barnes et al. (2009) found high cooccurrence of alcohol problems and problem gambling among young males.

Earlier onset of gambling has been found to increase the risk of later problem gambling among young people (Dowling et al., 2017) and older adults (Burge et al., 2004; Volberg et al., 2010). Lynch et al. (2004) demonstrated that adolescent gambling onset was associated with substance use disorders and psychiatric problems in young adulthood. Problem gambling among adolescents and young adults has been associated with harms in several domains including psychological and physical health, relationships, financial difficulties, and legal and criminal consequences (Blinn-Pike et al., 2010; Dowling et al., 2017; Public Health England, 2021; Riley et al., 2021; Wardle et al., 2019). Further public health concern arises from the economic burden of dealing with the costs of harmful gambling, estimated at more than £1 billion annually in England (Public Health England, 2021).

Turning to examine the association between the gambling industry and sport, the marketing of gambling in sport tends to be multi-dimensional, including shirt sponsorship, in-stadium banners, match commentary as well as television commercial breaks (Lopez-Gonzalez et al., 2018). For football in particular, advertising is embedded in sponsorship, match commentary, stadium banners, replica shirts, as well as TV ads “saturated” with gambling (Cassidy & Ovenden, 2017). Columb et al. (2020) documented that 75% of sports matches shown on television in Ireland had gambling advertisements. A qualitative study revealed that 18–35-year-old males were targeted in promotion and incentivisation for gambling, including embedding of sports betting in their social network (Deans et al., 2017a, 2017b). The content of gambling advertisements has been found to have a vast overrepresentation of male characters (Lopez-Gonzalez et al., 2018; Milner et al., 2013), and Thomas et al. (2015) found depictions of male camaraderie to be central in gambling marketing through social media. In a meta-analysis, Bouguettaya et al. (2020) report a positive association between gambling advertising and gambling behaviour, while also finding evidence of a dose–response relationship for advertising and some forms of gambling.

In light of the extensive targeting of sport by gambling advertisers, there is also evidence that participants in sport gamble more: male athletes have been found to have gambled more frequently (Huang et al., 2007; Martin et al., 2016; Vinberg et al., 2020), were more likely to problem gamble (Håkansson et al., 2021; Vinberg et al., 2020), and recreational athletes were found to have gambled more than elite athletes (Martin et al., 2016). Ellenbogen et al. (2008) found that male athletes in higher profile sports such as football and basketball were more likely to report problem gambling compared to participants in sports such as volleyball, while male team sport participants were more likely encounter a problem from gambling compared to males in individual sports (with the exception of golf). Nelson et al. (2007) found that both sports fans and sports participants gambled more frequent than non-fans and participants, after controlling for individual characteristics, including sex. A study by Gavriel-Fried et al. (2015) revealed that for young males and females, involvement in organised competitive sports was linked to a greater gambling frequency, compared non-organised competitive sports (such as gym workouts), though a link with problem gambling was only observed for males.

The evidence documented here indicates that gambling advertising may increase gambling behaviour, that sport has been targeted by advertisers as a domain to increase gambling behaviour, and that sports participants are more likely to gamble. However, the literature suggests this is not uniformly the case, with males and team sport participants overrepresented in gambling behaviour, compared to females and those engaged in individual sports. Some authors who found a relationship between athletes and gambling (e.g., Ellenbogen et al., 2008; Gavriel-Fried et al., 2015) point to an inherent competitiveness and higher risk appetite of sports players that may attract them to winning. However, this does not explain why only some players do while others do not (e.g., track and field athletes). For the purposes of this research, we offer an alternative explanation via the establishment and dissemination of gambling-based social norms among male team sport participants. The conceptual framework for this draws on social identity theory (Tajfel & Turner, 1979). Social identity is a core construct in the field of social psychology, and social norms that are theorised to arise from it have been extensively studied (Larimer & Neighbors, 2003). It proposes one’s social identity and membership of discrete social groups, is central to one’s self-concept (Tajfel, 1981). This is a two-stage process, where initially self-categorising as a group member leads to accentuation of similarities with other group members and differences with non-members. This is followed by self-enhancement, whereby individual behaviours and attitudes may adapt to be congruent with the perceived normative behaviours and attitudes of the group (Foster et al., 2014). It is through this process of social norm development that beliefs and behaviour of the individual group members are influenced (Cruwys et al., 2015), and group membership provides motivation to act in coherence with the groups’ perceived norms (Turner, 1991). Where a behaviour is perceived to be a norm within a group, members are more likely to engage in it, and younger adults maybe be particularly susceptible to the role of norms in influencing behaviour (Dishion & Tipsord, 2011; Tarrant, 2002). Furthermore, young sports team participants have been shown to have greater pro-social behaviour towards teammates (Bruner et al., 2014). Evidence of the role of social norms in alcohol use among young adults found perceived norms regarding alcohol consumption influences its social acceptability (Borsari & Carey, 2001). Moreover, Lisha and Sussman (2010), in a review of 34 studies, found that perceived norms of alcohol use was positively associated with consumption, and higher degrees of participation in sport was associated with greater alcohol use.

A small number of studies have examined the role of perceived norms in gambling behaviour. Larimer and Neighbors (2003) found that overestimates of gambling behaviour norms predicted self-reported gambling frequency and spending, while Foster et al. (2014) sought to replicate this earlier study and similarly found that perceived norms for gambling was associated with gambling behaviour. For this study, undertaking gambling is proposed to be a behavioural norm adopted among young males who participate in team sports at 17/18 years and at 20 years via group membership as underpinned by social identity theory. The empirical analysis of this paper, described in the next section of this paper, tests whether team sports participation over time is associated with gambling behaviours.

## Methods

### Design of Empirical Study

To inform the research questions, secondary data from two waves of the’98 Cohort of *Growing Up in Ireland* (GUI) are employed. GUI is a nationally representative longitudinal study of children and young people in Ireland which commenced in 2008, collecting extensive demographic, socioeconomic and health information on study participants. Sampling and data collection methodology has been reported elsewhere (Murray et al., 2010), but in brief, the’98 Cohort consisted of 8,568 families of nine-year-olds who were first interviewed between August 2007 and May 2008, with the sample selected based on the sampling frame of Irish primary schools. This research examines data from the two most recent waves from this cohort, waves 3 and 4. Wave 3 was conducted in 2015–16 when the young people were 17/18 years, capturing 6,216 participants. Wave 4 occurred throughout 2018/19 when the young people were 20 years, collecting information on 5,190 participants, representing 61% of the original 9-year-old sample (O’Mahony et al., 2021). In person interviews were conducted with both the young person and their primary caregiver through computer-assisted personal interviews (CAPI). Further data on topics that may be considered sensitive was collected through the self-complete questionnaire, administered using Computer-Assisted Self Interview (CASI). GUI is made available to researchers in the form of secondary data, accessible as anonymised research microdata files upon application and approval from the Central Statistics Office in Ireland.

### Measures

#### Dependent Variables

##### Engaging in Online Gambling

The 20-year-olds were asked in the ‘Internet and Technology use’ section of the self-complete questionnaire if they *use the internet* for 16 different activities, where “virtual casino/placing bets” was an option for which they could select. Where this option was selected, this is coded as = 1 for a binary variable indicating the young person engaged in online gambling, and zero otherwise.

##### ‘Regularly’ Gambling

In the ‘Smoking, Alcohol and Drugs’ section of the self-complete questionnaire, the 20-year-olds were asked how often they play three types of games “in person or online”. These were (1) lottery ticket, (2) casino tables or video games for money, and (3) other games for money, or bet on horses or sporting events or take part in other kinds of gambling for money. Seven frequency options were available as responses: a few times a week; once a week; once or twice a month; occasionally; a few times a year; and never. For the purposes of the research of this paper, the responses concerning frequency of undertaking these activities were collapsed to a binary variable for each option, delineated as equal to one where the frequency was ‘At least once a month’ (i.e., the participant selected either: a few times a week; once a week; once or twice a month), and all other options were marked as equal to zero. The dummy variable for gambling was ‘at least once a month’ in either (2) or (3), where this can be considered gambling regularly in either of these two gambling modes. Thus, the binary variable, ‘gamble regularly’ is a composite measure. Lottery ticket gambling was not included in the binary gambling outcome because of its lower prevalence among young adults compared to other forms and particularly sports betting (Mongan et al., 2022). Furthermore, research tends to find a far weaker relationship between lottery gambling behaviour and problem gambling (Griffiths, 2016).

#### Exposure of Interest—Sports Participation

To explore the application of social identity theory in the context of gambling behaviour among young people and participation in team sports, we are interested in the survey questions which inquire as to whether participants were engaged in team sports at the time of data collection, and for the length of time that they were engaged in it. At Wave 4, the 20-year-olds were asked in the main survey about activities they engaged in regularly, including a category for ‘participating in sport (with others)’ (response option: yes/no). The next category option was ‘participating in individual sport’, providing a delineation between group and individual participation in sports. In the main survey of Wave 3, the 17/18-year-olds were asked about activities they had taken part in over the last year, and among the response options the category ‘Sports clubs/teams’ was included.

The response options to the two questions concerned with team sports participation from Waves 3 and 4 were combined to develop a four-category derived variable ‘team sports participation’, with the following categories, where the respondent reported:

1. Team sports participation at both 20 years and 17/18 years
2. Team sports participation at age 20, but not aged 17
3. No team sports participation at age 20, but participated in team sports at age 17
4. No team sports participation at either wave.

We hypothesise that more prolonged and more recent exposure to team sports would have the largest effect of gambling behaviour since the development within a social group identity such as a sports group is partly a function of time. For example, where a young person plays with a local soccer team at 17, but discontinues, so that they are not participating at 20, the likelihood that behaviours of the group (such as gambling) will influence that young person’s behaviours is lower compared to where they are in the group through to 20 years.

#### Control Variables

Evidence of a relationship between engagement in team sports and gambling behaviour may be confounded by several factors which are included in the study as a set of control variables. These include sex, socioeconomic, and behavioural factors recorded when the young person was 20 years old. Their principal economic status as categorised into three categories: in education; in employment; not in education, employment, or training (NEET). Whether the young person is residing outside the family home at 20 years is controlled for. The structure of the young person’s household background is also included as a control: which includes whether they are from a one-parent household, the social class categorisation of their parent, and the highest educational attainment of their parent. Health-related risk behaviours included as controls include smoking, frequency of alcohol consumption, and having reported ever using cannabis. At 20 years, the young person is asked about their willingness to take risks, on a scale of 0–10 where 0 indicates ‘unwilling to take risks’, and 10 means ‘fully prepared to take risks’, which is included as a control. The study also considers the possibility that engagement in any sport, be that team or individual sports, may be associated with gambling, or that engagement in team activities, sporting or otherwise, may explain some gambling behaviour. As such, this study accounts for whether the respondent reports they undertake individual sports, or other group activities. Finally, further exploiting the longitudinal nature of the data, the models of gambling behaviours at 20 years control for a variable available from Wave 3, for when the young person was 17/18 years, where the young person was asked whether they had participated in online gambling in that prior wave.

### Statistical Analysis

To answer the research questions of this paper, bivariate associations between participation in team sports at 20 years and sample characteristics were first conducted, using chi-squared tests and student t-tests. For multivariate analysis, team sports participation at 17/18 years and 20 years (Wave 3 and Wave 4) were combined, and logistic regression was used to analyse independent associations between participation in team sports and the sample characteristics.

Descriptive statistics for the complete sample are weighted for representativeness and to account for attrition of the sample across the waves (details of the population weighting procedure can be found in McNamara et al., (2021)). The sample used for estimation is that for complete cases, where there are complete observations for the outcome, the exposure and the included covariates for the waves investigated in the model. The analytical sample size is 4,645 for which there is complete information on all variables of interest. All analysis was performed using STATA 17, and the threshold for statistical significance is a p-value of p < 0.05.

## Results

**Fig. 1:**
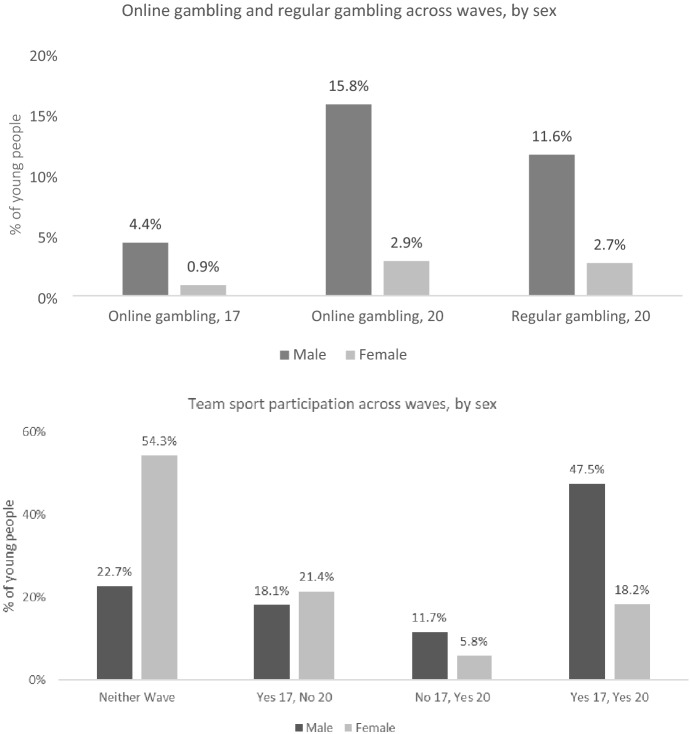
Participation in online gambling, regular gambling, and sports participation, by sex

**Fig. 2:**
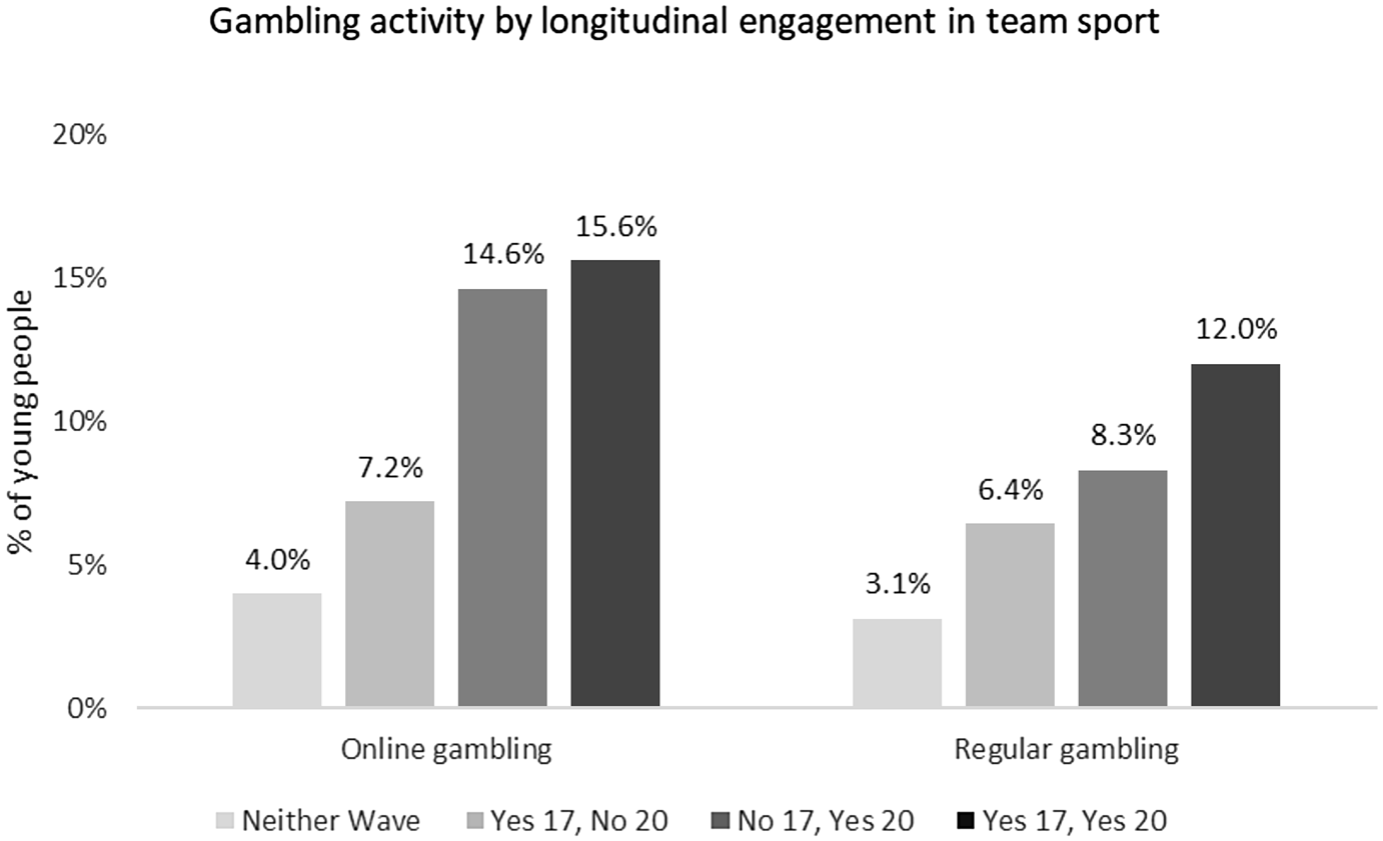
Gambling behaviours by team sports participation over time

Table 1 presents the descriptive statistics of the sample, weighted for representativeness (n = 4,571). At 20 years, 9.3% were engaging in online gambling (up from 2.6% at 17 years), and 7.2% were regularly gambling. Figure 1 breaks the gambling behaviours down by sex, showing a much greater prevalence for males. In terms of the exposure of interest, team sports participation over time, Table 1 demonstrates that about one third (32.9%) engage team sports at both time points, while 38.6% never participate in team sports in either wave, 19.7% dropped out of playing team sports between the ages of 17 and 20, and 8.8% took up team sport between 17 and 20 years. Figure 1 also shows the breakdown of team sports participation by sex, revealing that males were more likely to participate in team sports in both waves while females were more likely to be non-participants in team sports at both periods. Figure 2 shows greater levels of gambling activities were the young people have greater engagement in team sports over time.

**Table 1:** Summary statistics of the sample of *Growing Up in Ireland* study participants, weighted for representativeness

	Category	% / mean
*Outcome (at 20 years – wave 4)*		
Online gambling		9.3
Regularly gambling		7.2
*Exposure of interest (at 17/18 years (wave 3) and 20 years (wave 4))*		
Team sport participation	No 17, No 20	38.6
	Yes 17, No 20	19.7
	No 17, Yes 20	8.8
	Yes 17, Yes 20	32.9
*Covariates (measured at 20 years, wave 4, unless noted otherwise)*		
Sex	Male	50.1
	Female	49.9
Principal Economic Status	In Education	63.5
	Employment	31.4
	NEET	5.1
Living at non-parental address	Yes	32.7
Household type	One-parent	23.2
	Two-parent	76.8
Parent social class	Professional/ managerial	43.5
	Non-manual/skilled	33.5
	Semi-skilled/unskilled	13.0
	Lower/other	10.0
Parent highest education attainment	Degree	19.7
	Diploma/certificate	18.1
	Upper secondary	41.6
	Lower secondary or less	20.7
Smoking	Never	61.4
	Daily/occasionally	38.6
Frequency of alcohol consumption	Monthly or less	25.0
	2–4 Times per month	49.7
	At least weekly	25.4
Ever smoked cannabis		62.0
Risk appetite score (mean)		6.6
Online gambling at 17/18 years (wave 3)		2.6
Participates in individual’s sport (20 years)		29.1
Participates in other group activities (20 years)		9.3
Sample size: 4571

Table 1 reports that at 20 years, 29.1% report undertaking individual sports, and 9.3% were involved in other group activities. The weighted sample has an even sex split, the majority at 20 years are in education, one-in-three live outside the parental home, a quarter drink alcohol weekly, two in five reported smoking on an occasional or daily basis, and 62% reported that they had smoked cannabis.

The results displayed in Table 2 demonstrate a higher estimated odds of both online and regularly gambling where a young person reports having played team sports at both 20 years and 17 years, compared to where a young person never reports participation in team sports. For the pooled sample, the fully-adjusted model estimates that a 20-year-old who plays team sports both at 20 years and at 17 years has a 2.44 higher odds of online gambling, than the reference category of no engagement in team sports. The magnitude of the estimated odds of 1.97 is slightly lower for the comparison of the reference category and the category for which a young person did not report playing team sport at 17 but does report team sport at 20, than that of playing in both waves. In the fully adjusted model, there is no statistical difference for online gambling between never playing sports and for those who played sport at 17 years but had given it up by 20 years. Where the modelling is applied to the male only sample, the magnitude of the estimates is larger, where a 3.8 higher odds of online gambling is estimated among males who engaged in team sports in both waves, compared to never playing team sports. In terms of other covariates, in the pooled sample, being male has a strong positive association with online gambling at 20 years, as does having the least level of educational attainment, a higher risk appetite score, smoking, and previously reporting online gambling at 17 years. Engaging in other group activities has a statistically significant lower estimated odds for online gambling.

**Table 2:** Estimation results of factors associated with online gambling and regularly gambling at 20 years old

Outcome	Category	Online gambling				Regularly gambling			
		Pooled sample^†^			Male only	Pooled sample^†^			Male only
		(1)	(2)	(3)	(3)	(1)	(2)	(3)	(3)
Team Sport	Yes 17, No 20	1.44 +	1.42 +	1.35	1.85*	1.51 +	1.48 +	1.45	1.73 +
(ref. neither wave)	No 17, Yes 20	2.00**	2.13**	1.97**	3.21***	1.86*	2.04**	1.96*	2.80**
	Yes 17, Yes 20	2.62***	2.63***	2.44***	3.80***	3.08***	3.12***	2.99***	4.02***
Sex	Male	6.34***	5.65***	5.67***	−	5.58***	4.81***	4.84***	−
PES (ref. education)	Employment	1.27 +	1.16	1.66	1.14	1.39*	1.38*	1.38*	1.29 +
	NEET	1.71 +	1.61	1.64 +	1.67	1.63	1.79 +	1.85 +	1.35
Non-parent address	Yes	0.93	0.87	0.88	0.9	0.8	0.75*	0.76*	0.8
Household type	Two-parent	1.21	1.23	1.24	1.19	1.11	1.1	1.1	1.15
SES (ref. Prof/man)	NonM/Skill	0.85	0.83	0.82	0.88	0.91	0.92	0.92	0.93
	Semi/Unskill	1.24	1.16	1.19	1.1	1.37	1.41	1.44	1.11
	Lower/Other	0.66	0.65	0.67	0.7	1.29	1.4	1.43	1.38
PCG Education	Dip/Cert	1.19	1.18	1.17	1.08	1.12	1.1	1.09	1.23
(ref. Degree)	Upper 2nd	1.21	1.26	1.24	1.13	1.05	1.09	1.05	1.04
	Lower 2nd	1.79**	1.85**	1.85**	1.60 +	1.29	1.28	1.27	1.23
Smoking	Daily/Occas		1.39**	1.38*	1.35*		1.01	0.99	1.39 +
Alcohol	2–4 times p.m		0.85	0.87	1.04		1.71**	1.72**	2.12**
(ref. Monthly or less)	At least weekly		1.21	1.23	1.46 +		2.33***	2.34***	2.96***
Ever smoked cannabis			1.06	1.05	1.01		1.42*	1.40*	1.39 +
Risk appetite			1.09**	1.09**	1.07 +		1.04	1.05	1.08 +
W3 Online gambling	Yes		3.05***	3.05***	3.05***		3.08***	3.10***	3.37***
W4 Individual sport	Yes			1.25 +	1.25 +			1.04	0.99
W4 Other group activities	Yes			0.47**	0.49*			0.40**	0.39**
Log likelihood		−1139.69	−1112.38	−1105.89	−864.02	−962.75	−937.57	−932.18	723.91
Pseudo R2		13.03%	15.12%	15.61%	6.79%	12.37%	14.66%	15.15%	8.31%

***p < 0.001 **p < 0.01 *p < 0.05 + *p* < 0.10 denote statistical significance

^†^The sample comprises of 2203 males and 2442 females, providing a total pooled sample of 4645 study participants (unweighted). Estimates are presented as odds ratios

Turning to the estimation results for *regularly gambling*, for the pooled sample, and, the fully adjusted model, a 20-year-old who played team sports both at 20 and at 17 years has a 2.99 higher odds of regularly gambling, than the reference category of no engagement in team sports. For the male only sample, a 4.02 higher odds of regularly gambling is estimated for those who played sport in both waves compared to the base group. For the other covariates, while there are several similar associations between covariates estimated for regularly gambling and online gambling e.g., a statistically greater odds reported for males and those reporting prior engagement with online gambling at age 17, there are also slightly different statistical associations with regular gambling as unveiled in Table 2. Being in employment, compared to in education, is associated with a higher odds of engagement with frequently gambling, while living at a non-parental address is estimated to have a lower odds of gambling compared to living with parents. More regular alcohol consumption and cannabis use is associated with regularly gambling.

## Discussion

This study documents almost a four-fold increase in engagement in online gambling among a nationally representative cohort of young people in Ireland between the ages of 17/18 and 20 years. The increase is driven by males, where the prevalence of gambling rose from 4 to 16%. For the outcome of gambling regularly, at aged 20 years, males (12% prevalence) were far more likely to do so than females (3%). A statistically significant positive association is uncovered between playing team sports and engagement in regular gambling as well as online gambling behaviour, independent of socio-demographic and other risk factors *for males* but *not for females*. The findings of the research provide support for the hypothesis of a dose–response like effect *for males*, where a longer period of participation in team sports is associated with a higher likelihood of engaging in gambling behaviour compared to shorter periods. Additionally, those engaged in team sports for a shorter period are more likely to gamble compared to those who do not participate in team sports at all. The estimation results also demonstrate that more contemporaneous team sport participation has a greater association with gambling, where, playing team sports at 20 years is more strongly associated with online and regularly gambling at 20 years. The size and strength of the model estimates also indicate that team sports participation is a stronger predictor of gambling behaviours than prior gambling behaviour (at 17 years). As such, these results may be interpreted as supportive of the hypothesis that the establishment and dissemination of social norms around gambling among young males engaged in team sports plays a role in the higher prevalence of both online gambling behaviour and regular gambling (whether online or in more traditional modes) among males. While previous studies have established that young males are more likely to gamble than young females, and that participation in sport may be linked to a greater likelihood of gambling behaviour, this is the first study to offer an explanation that exposure to a team sport environment may play a role in increasing the likelihood in gambling among team sport participants. Furthermore, in employing a data from nationally representative prospective longitudinal cohort, this finding provides a strong platform to further investigate the influence of the team sports environment on future gambling behaviours among young males.

### Policy and Research Implications

The findings have implications for policy concerned with gambling behaviour. A core element of gambling policy has included youth gambling education initiatives (Schalkwyk et al., 2022). In the UK, teaching about the risks associated with gambling is a statutory requirement for all state-funded schools (Department of Education, 2019). This is not the case in Ireland, the study setting, although the proposed Gambling Regulation Bill, advises the establishment of a Gambling Regulatory Authority of Ireland under which public education and awareness programmes, and community intervention for gambling addiction, is proposed. The findings from the research of this paper suggests that there should be a targeted focus on higher risk groups of the population such as sports teams. Sports clubs, university, and college sports facilities could also provide a space in which education resources, materials, and information provision on gambling could be effective. Education and awareness campaigns could be run through colleges, youth clubs and camps. This paper acknowledges that some progress has been made on this area already, for example the Gaelic Players Association of the Gaelic Athletic Association published gambling guidelines for players in 2016 (Kelly et al., 2019), and in 2018 banned sponsorship deals with the gambling industry. Recently, the soccer Football Association of Ireland decided against partnering with a gambling company as its main sponsor, though there remains gambling sponsorship among soccer and rugby teams in Ireland, the UK and throughout Europe. More extremely, legislation could be introduced to restrict gambling advertising in sports. The European Parliament (2019) has published a policy options paper on harmful internet use, explicitly including online gambling addiction, and has identified dissemination and promotion of research on the risks of online gambling behaviour as a core policy option.

We suggest that there may be a need for correcting misperceptions of norms in gambling behaviour through social norm marketing and media campaigns which provide more accurate information on gambling rates. Using the results of this study, it can be highlighted that 80% of 20-year-olds do not gamble regularly. This may be contrasted with the findings from a survey of inter-county Gaelic football players in Ireland which reported that 80% of participants *believed* their teammates gambled at least weekly (Kelly et al., 2019). It has been argued that social norm campaigns among youth groups could create positive peer pressure which leads to behavioural change (Engwall et al., 2004). Sports clubs could develop policies and guidelines around gambling, and coaching staff and athletes could receive specialised education about identifying gambling harms and signposting to gambling interventions (Vinberg et al., 2020).

This important subject matter requires additional research scrutiny. Another wave of *Growing Up in Ireland* data will interview this cohort in 2023 where the young people will be 25 years, a peak age for problem gambling. Further research could examine whether the gambling and team sport relationship remains stable over time, and three waves of data on these issues could permit greater causal inference. Studies could examine whether there are sub-clinical effects of early onset gambling behaviour, and research from other jurisdictions could test the theory applied in this study to examine the generalisability of the findings. This study could not differentiate between the various types of team sports young adults participate in, and there could be important differences across different types of sports which merits scholarly investigation. Qualitative research could be undertaken in this area to understand the large sex differences observed, and it has been noted that increasingly gambling adverts have been oriented towards young women for which trends must be studied.

### Strengths and Limitations

This paper offers a novel theoretical approach to the study of gambling behaviours using social identity theory to explain the influence of sports team participation and engagement with gambling among young males. The study benefits from nationally representative data with a large sample of young people to test this theory. The longitudinal design of the dataset shows the development of the exposure of interest, sports team participation, and the gambling outcomes of study over a four-year period. The rich data source contains a range of socioeconomic and behaviour variables which allow the models to control for range of relevant life circumstances and factors which could confound the associations of interest.

Some limitations of the work must also be acknowledged. The waves of the *Growing Up in Ireland* dataset utilised in this paper do not contain a validated measure of problem gambling or data on gambling spend by participants. This could be investigated in future waves. We also note that there were slight differences in the wording of questions concerning participation in team sport at waves three and four which required harmonisation. Finally, the estimated results are based on observational data, which may be interpretated as associations, although we purport that the longitudinal analysis provides some epistemic weight.

## Conclusion

The engagement with online gambling quadrupled among males in Ireland from the ages of 17 to 20 years. Over time, participation in team sports, is revealed to have a dose–response like association with online and regularly gambling for young males. The findings of the empirical analysis provide support for the theory that the social norms that develop within the young male adult sports team environment may play a role in generating increased gambling behaviour, in both online and traditional modes, among team members. As such, team and sports environments also provide suitable forums for which gambling awareness and behaviours can be discussed and addressed. The findings of this research provide evidence to inform the development of policies and legislation in the area of online and regular gambling, and its links to young people and sports participation.

## Data Availability

Results in this report are based on analyses of data from Research Microdata Files provided by the Central Statistics Office (CSO). Growing Up in Ireland (GUI) is funded by the Department of Children and Youth Affairs (DCYA). It is managed by DCYA in association with the Central Statistics Office (CSO). Neither the CSO nor DCYA take any responsibility for the views expressed or the outputs generated from these analyses.
